# Supplementary material for: A Genetic Trap in Yeast for Inhibitors of SARS-CoV-2 Main Protease
Source: mSystems. 2021 Nov 23;6(6):e01087-21. doi: 10.1128/mSystems.01087-21 (PMC8609969; doi:10.1128/mSystems.01087-21)
Supplement: TEXT S1 [file msystems.01087-21-t0001.docx]

**File S1 Strain and plasmid construction and sequences**

***Strain construction***

HA_SC_1352control

Deletions of *PDR1*, *PDR3* and *SNQ2* in the parental strain 1352-Y13363 were confirmed by PCR. *MET17* was amplified from the CENPK2 background using primers Met17_pdr3_int_F and Met17_pdr3_int_R, this fragment serves both as a recombination marker and ligation fragment due to the presence of two *Bam* HI sites in the tails of the forward and reverse primer. The *MET17* fragment was transformed into the *PDR3* locus of strain 1352-Y13363 as described previously (R.D. Gietz RD, R.A. Woods, Meth Enzymol 350:87-96, 2002, https://doi.org/10.1016/s0076-6879(02)50957-5), hence replacing the *URA3* marker and allowing the use of methionine-controlled promoters and *URA3* based plasmids.

This strain was given the designation HA_SC_1352control. HA_SC_1352control_RED is a fluorescent derivative of HA_SC_1352control, in which an mCherry tag was amplified from plasmid YEP_CHERRY_HIS3 (E. Bilsland *et al.* Open Biol 3:120158, 2013, https://doi.org/10.1098/rsob.120158) using primer pair Flurotag_marker_F and Flurotag_marker_R and integrated in the *HIS3* locus.

HA_SC_Met17_Mpro

An artificial fragment carrying the SARS-COV2 major protease (Mpro, ACCESSION #NC_045512) with an added start and stop codon driven by the *Pichia GAP* promoter and terminated by the *FUM1* terminator (K.A. Curran *et al.* Metab Eng 19:88-97, 2013, https://doi.org/10.1016/j.ymben.2013.07.001) was optimized for expression in *S. cerevisiae* and synthesized (GenScript, Piscataway, NJ) then ligated into the *Bam* HI / *Bgl* II site of pPICZ(alpha) A to create plasmid Mpro_pPICZa. Mpro_pPICZa was digested with *Bam* HI and dephosphorylated. Subsequently the above amplified *MET17* fragment was digested with *Bam* HI and ligated to Mpro_pPICZa. A fragment carrying *MET17* ligated to Mpro was amplified from the ligation reaction using primers Mpro_met17_pdr3_int_F and Met17_pdr3_int_R and integrated into the *PDR3* locus of strain 1352-Y13363 to create strain HA_SC_Met17_Mpro (final sequence can be found in Supplementary sequences S1). HA_SC_Met17_Mpro_RED is a fluorescent derivative of HA_SC_Met17_Mpro and was constructed same as HA_SC_1352control_RED. All primers used for strain constructions are listed in Table S1.

***Plasmid construction***

pCM188-GAL1

Empty backbone for galactose driven toxin expression experiments was constructed by ligating the *GAL1* promoter flanked by *Eco* R1 and *Bgl* II into pCM188 (E. Garí *et al.* Yeast 13:837-848, 1997, https://doi.org/10.1002/(SICI)1097-0061(199707)13:9<837::AID-YEA145>3.0.CO;2-T) digested by *Eco* R1 and *Bam* HI.

pCM188-MET3

Empty backbone for methionine-controlled toxin expression experiments was constructed by ligating the *MET3* promoter flanked by *Eco* R1 and *Bam* HI into pCM188 (E. Garí *et al.* Yeast **13:**837-848, 1997, https://doi.org/10.1002/(SICI)1097-0061(199707) digested by *Eco* R1 and *Bam* HI.

PSM (positive selection module)

A chimeric synthetic construct expressing the 41 C‑terminal amino acid residues (aa) Leu42 to Trp81 of MazE with an added ATG codon preceding the Leu42 codon, followed by a GGVKLQSGS aa linker sequence containing the Mpro cleavage site VKLQS, joint N-terminally to the full aa sequence of MazF, codon optimized for *S. cerevisiae*, and synthesized (GenScript, Piscataway, NJ). This was then ligated into the *Bam* HI / *Pst* I site of pCM188 to create PSMv3. The full sequence of the MazEF chimera can be found below in this file). PSMv3-GAL was created by ligating the *GAL1* promoter fragment used above to the *Eco* R1 / *Bam* HI sites of PSMv3. PSMv4 was created by ligating the *MET3* promoter fragment used above to *Eco* R1 / *Bam* HI sites of PSMv3.

***Sequence PSMv3-Gal Genbank format***

LOCUS Exported 6774 bp DNA circular SYN 27-AUG-2021

DEFINITION synthetic circular DNA.

ACCESSION .

VERSION .

KEYWORDS .

SOURCE synthetic DNA construct

ORGANISM synthetic DNA construct

REFERENCE 1 (bases 1 to 6774)

AUTHORS Hanna Alalam

TITLE Direct Submission

JOURNAL Exported Friday, Aug 27, 2021 from SnapGene Viewer 5.3.0

https://www.snapgene.com

FEATURES Location/Qualifiers

source 1..6774

/organism="recombinant plasmid"

/mol_type="other DNA"

promoter 7..459

/label=Gal1p

/note="gal1 promoter"

CDS 466..954

/codon_start=1

/label=MazEF fusion-Mpro

/translation="MLIIEPVRKEPVFTLAELVNDITPENLHENIDWGEPKDKEVWGGV

KLQSGSMVSRYVPDMGDLIWVDFDPTKGSEQAGHRPAVVLSPFMYNNKTGMCLCVPCTT

QSKGYPFEVVLSGQERDGVALADQVKSIAWRARGATKKGTVAPEELQLIKAKINVLIG"

CDS 466..591

/codon_start=1

/label=mazE C terminal

/translation="MLIIEPVRKEPVFTLAELVNDITPENLHENIDWGEPKDKEVW"

misc_difference 592..597

/label=Flexibility linker

misc_feature 598..612

/label=SARS-CoV-2 Mpro cleavage site

misc_feature 613..618

/label=Flexibility linker

CDS 619..954

/codon_start=1

/label=mazF

/translation="MVSRYVPDMGDLIWVDFDPTKGSEQAGHRPAVVLSPFMYNNKTGM

CLCVPCTTQSKGYPFEVVLSGQERDGVALADQVKSIAWRARGATKKGTVAPEELQLIKA

KINVLIG"

misc_feature 960

/label=MCS

terminator 961..1222

/label=CYC1 terminator

/label=CYC1\terminator

rep_origin 1638..2231

/label=pMB1 ori

/label=pMB1\ori

CDS complement(2402..3262)

/codon_start=1

/label=ampR

/translation="MSIQHFRVALIPFFAAFCLPVFAHPETLVKVKDAEDQLGARVGYI

ELDLNSGKILESFRPEERFPMMSTFKVLLCGAVLSRIDAGQEQLGRRIHYSQNDLVEYS

PVTEKHLTDGMTVRELCSAAITMSDNTAANLLLTTIGGPKELTAFLHNMGDHVTRLDRW

EPELNEAIPNDERDTTMPVAMATTLRKLLTGELLTLASRQQLIDWMEADKVAGPLLRSA

LPAGWFIADKSGAGERGSRGIIAALGPDGKPSRIVVIYTTGSQATMDERNRQIAEIGAS

LIKHW"

CDS 3699..4501

/codon_start=1

/label=URA3

/translation="MSKATYKERAATHPSPVAAKLFNIMHEKQTNLCASLDVRTTKELL

ELVEALGPKICLLKTHVDILTDFSMEGTVKPLKALSAKYNFLLFEDRKFADIGNTVKLQ

YSAGVYRIAEWADITNAHGVVGPGIVSGLKQAAEEVTKEPRGLLMLAELSCKGSLSTGE

YTKGTVDIAKSDKDFVIGFIAQRDMGGRDEGYDWLIMTPGVGLDDKGDALGQQYRTVDD

VVSTGSDIIIVGRGLFAKGRDAKVEGERYRKAGWEAYLRRCGQQN"

rep_origin 4839..5583

/label=ARS1

rep_origin 5584..6560

/label=CEN 4

/label=CEN\4

ORIGIN

1 gaattcagta cggattagaa gccgccgagc gggcgacagc cctccgacgg aagactctcc

61 tccgtgcgtc ctcgtcttca ccggtcgcgt tcctgaaacg cagatgtgcc tcgcgccgca

121 ctgctccgaa caataaagat tctacaatac tagcttttat ggttatgaag aggaaaaatt

181 ggcagtaacc tggccccaca aaccttcaaa ttaacgaatc aaattaacaa ccataggatg

241 ataatgcgat tagtttttta gccttatttc tggggtaatt aatcagcgaa gcgatgattt

301 ttgatctatt aacagatata taaatggaaa agctgcataa ccactttaac taatactttc

361 aacattttca gtttgtatta cttcttattc aaatgtcata aaagtatcaa caaaaaattg

421 ttaatatacc tctatacttt aacgtcaagg agaaaaaaca gatccatgtt gattattgaa

481 ccagttagaa aagaaccagt ttttactttg gctgaattgg ttaatgatat tactccagaa

541 aatttgcatg aaaatattga ttggggtgaa ccaaaagata aagaagtttg gggtggtgtt

601 aaattgcaat ctggttctat ggtttctaga tatgttccag atatgggtga tttgatttgg

661 gttgattttg atccaactaa aggttctgag caagctggtc atagaccagc tgttgttttg

721 tctccattta tgtataataa taaaactggt atgtgtttgt gtgttccatg tactactcaa

781 tctaaaggtt atccatttga agttgttttg tctggtcaag aaagagatgg tgttgctttg

841 gctgatcaag ttaaatctat tgcttggaga gctagaggtg ctactaaaaa aggtactgtt

901 gctccagaag aattgcaatt gattaaagct aaaattaatg ttttgattgg ttaactgcag

961 gagggccgca tcatgtaatt agttatgtca cgcttacatt cacgccctcc ccccacatcc

1021 gctctaaccg aaaaggaagg agttagacaa cctgaagtct aggtccctat ttattttttt

1081 atagttatgt tagtattaag aacgttattt atatttcaaa tttttctttt ttttctgtac

1141 agacgcgtgt acgcatgtaa cattatactg aaaaccttgc ttgagaaggt tttgggacgc

1201 tcgaaggctt taatttgcgg ccaagcttgg cgtaatcatg gtcatagctg tttcctgtgt

1261 gaaattgtta tccgctcaca attccacaca acatacgagc cggaagcata aagtgtaaag

1321 cctggggtgc ctaatgagtg agctaactca cattaattgc gttgcgctca ctgcccgctt

1381 tccagtcggg aaacctgtcg tgccagctgc attaatgaat cggccaacgc gcggggagag

1441 gcggtttgcg tattgggcgc tcttccgctt cctcgctcac tgactcgctg cgctcggtcg

1501 ttcggctgcg gcgagcggta tcagctcact caaaggcggt aatacggtta tccacagaat

1561 caggggataa cgcaggaaag aacatgtgag caaaaggcca gcaaaaggcc aggaaccgta

1621 aaaaggccgc gttgctggcg tttttccata ggctccgccc ccctgacgag catcacaaaa

1681 atcgacgctc aagtcagagg tggcgaaacc cgacaggact ataaagatac caggcgtttc

1741 cccctggaag ctccctcgtg cgctctcctg ttccgaccct gccgcttacc ggatacctgt

1801 ccgcctttct cccttcggga agcgtggcgc tttctcatag ctcacgctgt aggtatctca

1861 gttcggtgta ggtcgttcgc tccaagctgg gctgtgtgca cgaacccccc gttcagcccg

1921 accgctgcgc cttatccggt aactatcgtc ttgagtccaa cccggtaaga cacgacttat

1981 cgccactggc agcagccact ggtaacagga ttagcagagc gaggtatgta ggcggtgcta

2041 cagagttctt gaagtggtgg cctaactacg gctacactag aaggacagta tttggtatct

2101 gcgctctgct gaagccagtt accttcggaa aaagagttgg tagctcttga tccggcaaac

2161 aaaccaccgc tggtagcggt ggtttttttg tttgcaagca gcagattacg cgcagaaaaa

2221 aaggatctca agaagatcct ttgatctttt ctacggggtc tgacgctcag tggaacgaaa

2281 actcacgtta agggattttg gtcatgagat tatcaaaaag gatcttcacc tagatccttt

2341 taaattaaaa atgaagtttt aaatcaatct aaagtatata tgagtaaact tggtctgaca

2401 gttaccaatg cttaatcagt gaggcaccta tctcagcgat ctgtctattt cgttcatcca

2461 tagttgcctg actccccgtc gtgtagataa ctacgatacg ggagggctta ccatctggcc

2521 ccagtgctgc aatgataccg cgagacccac gctcaccggc tccagattta tcagcaataa

2581 accagccagc cggaagggcc gagcgcagaa gtggtcctgc aactttatcc gcctccatcc

2641 agtctattaa ttgttgccgg gaagctagag taagtagttc gccagttaat agtttgcgca

2701 acgttgttgc cattgctaca ggcatcgtgg tgtcacgctc gtcgtttggt atggcttcat

2761 tcagctccgg ttcccaacga tcaaggcgag ttacatgatc ccccatgttg tgcaaaaaag

2821 cggttagctc cttcggtcct ccgatcgttg tcagaagtaa gttggccgca gtgttatcac

2881 tcatggttat ggcagcactg cataattctc ttactgtcat gccatccgta agatgctttt

2941 ctgtgactgg tgagtactca accaagtcat tctgagaata gtgtatgcgg cgaccgagtt

3001 gctcttgccc ggcgtcaata cgggataata ccgcgccaca tagcagaact ttaaaagtgc

3061 tcatcattgg aaaacgttct tcggggcgaa aactctcaag gatcttaccg ctgttgagat

3121 ccagttcgat gtaacccact cgtgcaccca actgatcttc agcatctttt actttcacca

3181 gcgtttctgg gtgagcaaaa acaggaaggc aaaatgccgc aaaaaaggga ataagggcga

3241 cacggaaatg ttgaatactc atactcttcc tttttcaata ttattgaagc atttatcagg

3301 gttattgtct catgagcgga tacatatttg aatgtattta gaaaaataaa caaatagggg

3361 ttccgcgcac atttccccga aaagtgccac ctgacgtcta agaaaccatt attatcatga

3421 cattaaccta taaaaatagg cgtatcacga ggccctttcg tcttcaagaa ttagcttttc

3481 aattcaattc atcatttttt ttttattctt ttttttgatt tcggtttctt tgaaattttt

3541 ttgattcggt aatctccgaa cagaaggaag aacgaaggaa ggagcacaga cttagattgg

3601 tatatatacg catatgtagt gttgaagaaa catgaaattg cccagtattc ttaacccaac

3661 tgcacagaac aaaaacatgc aggaaacgaa gataaatcat gtcgaaagct acatataagg

3721 aacgtgctgc tactcatcct agtcctgttg ctgccaagct atttaatatc atgcacgaaa

3781 agcaaacaaa cttgtgtgct tcattggatg ttcgtaccac caaggaatta ctggagttag

3841 ttgaagcatt aggtcccaaa atttgtttac taaaaacaca tgtggatatc ttgactgatt

3901 tttccatgga gggcacagtt aagccgctaa aggcattatc cgccaagtac aattttttac

3961 tcttcgaaga cagaaaattt gctgacattg gtaatacagt caaattgcag tactctgcgg

4021 gtgtatacag aatagcagaa tgggcagaca ttacgaatgc acacggtgtg gtgggcccag

4081 gtattgttag cggtttgaag caggcggcag aagaagtaac aaaggaacct agaggccttt

4141 tgatgttagc agaattgtca tgcaagggct ccctatctac tggagaatat actaagggta

4201 ctgttgacat tgcgaagagc gacaaagatt ttgttatcgg ctttattgct caaagagaca

4261 tgggtggaag agatgaaggt tacgattggt tgattatgac acccggtgtg ggtttagatg

4321 acaagggaga cgcattgggt caacagtata gaaccgtgga tgatgtggtc tctacaggat

4381 ctgacattat tattgttgga agaggactat ttgcaaaggg aagggatgct aaggtagagg

4441 gtgaacgtta cagaaaagca ggctgggaag catatttgag aagatgcggc cagcaaaact

4501 aaaaaactgt attataagta aatgcatgta tactaaactc acaaattaga gcttcaattt

4561 aattatatca gttattaccc aattctcatg tttgacagct tatcatcgat cgtccaactg

4621 catggagatg agtcgtggca agaataccaa gagttcctcg gtttgccagt tattaaaaga

4681 ctcgtatttc caaaagactg caacatacta ctcagtgcag cttcacagaa acctcattcg

4741 tttattccct tgtttgattc agaagcaggt gggacaggtg aacttttgga ttggaactcg

4801 atttctgact gggttggaag gcaagagagc cccgagagct tacattttat gttagctggt

4861 ggactgacgc cagaaaatgt tggtgatgcg cttagattaa atggcgttat tggtgttgat

4921 gtaagcggag gtgtggagac aaatggtgta aaagactcta acaaaatagc aaatttcgtc

4981 aaaaatgcta agaaataggt tattactgag tagtatttat ttaagtattg tttgtgcact

5041 tgcctgcaag ccttttgaaa agcaagcata aaagatctaa acataaaatc tgtaaaataa

5101 caagatgtaa agataatgct aaatcatttg gctttttgat tgattgtaca ggaaaatata

5161 catcgcaggg ggttgacttt taccatttca ccgcaatgga atcaaacttg ttgaagagaa

5221 tgttcacagg cgcatacgct acaatgaccc gattcttgct agccttttct cggtcttgca

5281 aacaaccgcc ggcagcttag tatataaata cacatgtaca tacctctctc cgtatcctcg

5341 taatcatttt cttgtattta tcgtcttttc gctgtaaaaa ctttatcaca cttatctcaa

5401 atacacttat taaccgcttt tactattatc ttctacgctg acagtaatat caaacagtga

5461 cacatattaa acacagtggt ttctttgcat aaacaccatc agcctcaagt cgtcaagtaa

5521 agatttcgtg ttcatgcaga tagataacaa tctatatgtt gataattagc gttgcctcat

5581 caatgcgaga tccgtttaac cggaccctag tgcacttacc ccacgttcgg tccactgtgt

5641 gccgaacatg ctccttcact attttaacat gtggaattaa ttctcatgtt tgacagctta

5701 tcatcgaact ctaagaggtg atacttattt actgtaaaac tgtgacgata aaaccggaag

5761 gaagaataag aaaactcgaa ctgatctata atgcctattt tctgtaaaga gtttaagcta

5821 tgaaagcctc ggcattttgg ccgctcctag gtagtgcttt ttttccaagg acaaaacagt

5881 ttctttttct tgagcaggtt ttatgtttcg gtaatcataa acaataaata aattatttca

5941 tttatgttta aaaataaaaa ataaaaaagt attttaaatt tttaaaaaag ttgattataa

6001 gcatgtgacc ttttgcaagc aattaaattt tgcaatttgt gattttaggc aaaagttaca

6061 atttctggct cgtgtaatat atgtatgcta aagtgaactt ttacaaagtc gatatggact

6121 tagtcaaaag aaattttctt aaaaatatat agcactagcc aatttagcac ttctttatga

6181 gatatattat agactttatt aagccagatt tgtgtattat atgtatttac ccggcgaatc

6241 atggacatac attctgaaat aggtaatatt ctctatggtg agacagcata gataacctag

6301 gatacaagtt aaaagctagt actgttttgc agtaattttt ttctttttta taagaatgtt

6361 accacctaaa taagttataa agtcaatagt taagtttgat atttgattgt aaaataccgt

6421 aatatatttg catgatcaaa aggctcaatg ttgactagcc agcatgtcaa ccactatatt

6481 gatcaccgat atatggactt ccacaccaac tagtaatatg acaataaatt caagatattc

6541 ttcatgagaa tggcccagcg atatatgcgg tgtgaaatac cgcacagatg cgtaaggaga

6601 aaataccgca tcaggcgcca ttcgccattc aggctgcgca actgttggga agggcgatcg

6661 gtgcgggcct cttcgctatt acgccagctg gcgaaagggg gatgtgctgc aaggcgatta

6721 agttgggtaa cgccagggtt ttcccagtca cgacgttgta aaacgacggc cagt

//

***Sequence pCM188-MET3 Genbank format***

LOCUS Exported 6368 bp DNA circular SYN 27-AUG-2021

DEFINITION synthetic circular DNA.

ACCESSION .

VERSION .

KEYWORDS .

SOURCE synthetic DNA construct

ORGANISM synthetic DNA construct

REFERENCE 1 (bases 1 to 6368)

AUTHORS Hanna Alalam

TITLE Direct Submission

JOURNAL Exported Friday, Aug 27, 2021 from SnapGene Viewer 5.3.0

https://www.snapgene.com

FEATURES Location/Qualifiers

source 1..6368

/organism="recombinant plasmid"

/mol_type="other DNA"

promoter 7..500

/label=Met3p

misc_feature 502..554

/label=MCS

terminator 555..816

/label=CYC1 terminator

/label=CYC1\terminator

rep_origin 1232..1825

/label=pMB1 ori

/label=pMB1\ori

CDS complement(1996..2856)

/codon_start=1

/label=ampR

/translation="MSIQHFRVALIPFFAAFCLPVFAHPETLVKVKDAEDQLGARVGYI

ELDLNSGKILESFRPEERFPMMSTFKVLLCGAVLSRIDAGQEQLGRRIHYSQNDLVEYS

PVTEKHLTDGMTVRELCSAAITMSDNTAANLLLTTIGGPKELTAFLHNMGDHVTRLDRW

EPELNEAIPNDERDTTMPVAMATTLRKLLTGELLTLASRQQLIDWMEADKVAGPLLRSA

LPAGWFIADKSGAGERGSRGIIAALGPDGKPSRIVVIYTTGSQATMDERNRQIAEIGAS

LIKHW"

CDS 3293..4095

/codon_start=1

/label=URA3

/translation="MSKATYKERAATHPSPVAAKLFNIMHEKQTNLCASLDVRTTKELL

ELVEALGPKICLLKTHVDILTDFSMEGTVKPLKALSAKYNFLLFEDRKFADIGNTVKLQ

YSAGVYRIAEWADITNAHGVVGPGIVSGLKQAAEEVTKEPRGLLMLAELSCKGSLSTGE

YTKGTVDIAKSDKDFVIGFIAQRDMGGRDEGYDWLIMTPGVGLDDKGDALGQQYRTVDD

VVSTGSDIIIVGRGLFAKGRDAKVEGERYRKAGWEAYLRRCGQQN"

rep_origin 4433..5177

/label=ARS1

rep_origin 5178..6154

/label=CEN 4

/label=CEN\4

ORIGIN

1 gaattcttta gtactaacag agacttttgt cacaactaca tataagtgta caaatatagt

61 acagatatga cacacttgta gcgccaacgc gcatcctacg gattgctgac agaaaaaaag

121 gtcacgtgac cagaaaagtc acgtgtaatt ttgtaactca ccgcattcta gcggtccctg

181 tcgtgcacac tgcactcaac accataaacc ttagcaacct ccaaaggaaa tcaccgtata

241 acaaagccac agttttacaa cttagtctct tatgaagtta cttaccaatg agaaatagag

301 gctctttctc gagaaatatg aatatggata tatatatata tatatatata tatatatata

361 tatatgtaaa cttggttctt ttttagcttg tgatctctag cttgggtctc tctctgtcgt

421 aacagttgtg atatcgtttc ttaacaattg aaaaggaact aagaaagtat aataataaca

481 agaataaagt ataattaaca ggatccgttt aaacaggcct gttaacatcg atagcggccg

541 ctagggccct gcaggagggc cgcatcatgt aattagttat gtcacgctta cattcacgcc

601 ctccccccac atccgctcta accgaaaagg aaggagttag acaacctgaa gtctaggtcc

661 ctatttattt ttttatagtt atgttagtat taagaacgtt atttatattt caaatttttc

721 ttttttttct gtacagacgc gtgtacgcat gtaacattat actgaaaacc ttgcttgaga

781 aggttttggg acgctcgaag gctttaattt gcggccaagc ttggcgtaat catggtcata

841 gctgtttcct gtgtgaaatt gttatccgct cacaattcca cacaacatac gagccggaag

901 cataaagtgt aaagcctggg gtgcctaatg agtgagctaa ctcacattaa ttgcgttgcg

961 ctcactgccc gctttccagt cgggaaacct gtcgtgccag ctgcattaat gaatcggcca

1021 acgcgcgggg agaggcggtt tgcgtattgg gcgctcttcc gcttcctcgc tcactgactc

1081 gctgcgctcg gtcgttcggc tgcggcgagc ggtatcagct cactcaaagg cggtaatacg

1141 gttatccaca gaatcagggg ataacgcagg aaagaacatg tgagcaaaag gccagcaaaa

1201 ggccaggaac cgtaaaaagg ccgcgttgct ggcgtttttc cataggctcc gcccccctga

1261 cgagcatcac aaaaatcgac gctcaagtca gaggtggcga aacccgacag gactataaag

1321 ataccaggcg tttccccctg gaagctccct cgtgcgctct cctgttccga ccctgccgct

1381 taccggatac ctgtccgcct ttctcccttc gggaagcgtg gcgctttctc atagctcacg

1441 ctgtaggtat ctcagttcgg tgtaggtcgt tcgctccaag ctgggctgtg tgcacgaacc

1501 ccccgttcag cccgaccgct gcgccttatc cggtaactat cgtcttgagt ccaacccggt

1561 aagacacgac ttatcgccac tggcagcagc cactggtaac aggattagca gagcgaggta

1621 tgtaggcggt gctacagagt tcttgaagtg gtggcctaac tacggctaca ctagaaggac

1681 agtatttggt atctgcgctc tgctgaagcc agttaccttc ggaaaaagag ttggtagctc

1741 ttgatccggc aaacaaacca ccgctggtag cggtggtttt tttgtttgca agcagcagat

1801 tacgcgcaga aaaaaaggat ctcaagaaga tcctttgatc ttttctacgg ggtctgacgc

1861 tcagtggaac gaaaactcac gttaagggat tttggtcatg agattatcaa aaaggatctt

1921 cacctagatc cttttaaatt aaaaatgaag ttttaaatca atctaaagta tatatgagta

1981 aacttggtct gacagttacc aatgcttaat cagtgaggca cctatctcag cgatctgtct

2041 atttcgttca tccatagttg cctgactccc cgtcgtgtag ataactacga tacgggaggg

2101 cttaccatct ggccccagtg ctgcaatgat accgcgagac ccacgctcac cggctccaga

2161 tttatcagca ataaaccagc cagccggaag ggccgagcgc agaagtggtc ctgcaacttt

2221 atccgcctcc atccagtcta ttaattgttg ccgggaagct agagtaagta gttcgccagt

2281 taatagtttg cgcaacgttg ttgccattgc tacaggcatc gtggtgtcac gctcgtcgtt

2341 tggtatggct tcattcagct ccggttccca acgatcaagg cgagttacat gatcccccat

2401 gttgtgcaaa aaagcggtta gctccttcgg tcctccgatc gttgtcagaa gtaagttggc

2461 cgcagtgtta tcactcatgg ttatggcagc actgcataat tctcttactg tcatgccatc

2521 cgtaagatgc ttttctgtga ctggtgagta ctcaaccaag tcattctgag aatagtgtat

2581 gcggcgaccg agttgctctt gcccggcgtc aatacgggat aataccgcgc cacatagcag

2641 aactttaaaa gtgctcatca ttggaaaacg ttcttcgggg cgaaaactct caaggatctt

2701 accgctgttg agatccagtt cgatgtaacc cactcgtgca cccaactgat cttcagcatc

2761 ttttactttc accagcgttt ctgggtgagc aaaaacagga aggcaaaatg ccgcaaaaaa

2821 gggaataagg gcgacacgga aatgttgaat actcatactc ttcctttttc aatattattg

2881 aagcatttat cagggttatt gtctcatgag cggatacata tttgaatgta tttagaaaaa

2941 taaacaaata ggggttccgc gcacatttcc ccgaaaagtg ccacctgacg tctaagaaac

3001 cattattatc atgacattaa cctataaaaa taggcgtatc acgaggccct ttcgtcttca

3061 agaattagct tttcaattca attcatcatt ttttttttat tctttttttt gatttcggtt

3121 tctttgaaat ttttttgatt cggtaatctc cgaacagaag gaagaacgaa ggaaggagca

3181 cagacttaga ttggtatata tacgcatatg tagtgttgaa gaaacatgaa attgcccagt

3241 attcttaacc caactgcaca gaacaaaaac atgcaggaaa cgaagataaa tcatgtcgaa

3301 agctacatat aaggaacgtg ctgctactca tcctagtcct gttgctgcca agctatttaa

3361 tatcatgcac gaaaagcaaa caaacttgtg tgcttcattg gatgttcgta ccaccaagga

3421 attactggag ttagttgaag cattaggtcc caaaatttgt ttactaaaaa cacatgtgga

3481 tatcttgact gatttttcca tggagggcac agttaagccg ctaaaggcat tatccgccaa

3541 gtacaatttt ttactcttcg aagacagaaa atttgctgac attggtaata cagtcaaatt

3601 gcagtactct gcgggtgtat acagaatagc agaatgggca gacattacga atgcacacgg

3661 tgtggtgggc ccaggtattg ttagcggttt gaagcaggcg gcagaagaag taacaaagga

3721 acctagaggc cttttgatgt tagcagaatt gtcatgcaag ggctccctat ctactggaga

3781 atatactaag ggtactgttg acattgcgaa gagcgacaaa gattttgtta tcggctttat

3841 tgctcaaaga gacatgggtg gaagagatga aggttacgat tggttgatta tgacacccgg

3901 tgtgggttta gatgacaagg gagacgcatt gggtcaacag tatagaaccg tggatgatgt

3961 ggtctctaca ggatctgaca ttattattgt tggaagagga ctatttgcaa agggaaggga

4021 tgctaaggta gagggtgaac gttacagaaa agcaggctgg gaagcatatt tgagaagatg

4081 cggccagcaa aactaaaaaa ctgtattata agtaaatgca tgtatactaa actcacaaat

4141 tagagcttca atttaattat atcagttatt acccaattct catgtttgac agcttatcat

4201 cgatcgtcca actgcatgga gatgagtcgt ggcaagaata ccaagagttc ctcggtttgc

4261 cagttattaa aagactcgta tttccaaaag actgcaacat actactcagt gcagcttcac

4321 agaaacctca ttcgtttatt cccttgtttg attcagaagc aggtgggaca ggtgaacttt

4381 tggattggaa ctcgatttct gactgggttg gaaggcaaga gagccccgag agcttacatt

4441 ttatgttagc tggtggactg acgccagaaa atgttggtga tgcgcttaga ttaaatggcg

4501 ttattggtgt tgatgtaagc ggaggtgtgg agacaaatgg tgtaaaagac tctaacaaaa

4561 tagcaaattt cgtcaaaaat gctaagaaat aggttattac tgagtagtat ttatttaagt

4621 attgtttgtg cacttgcctg caagcctttt gaaaagcaag cataaaagat ctaaacataa

4681 aatctgtaaa ataacaagat gtaaagataa tgctaaatca tttggctttt tgattgattg

4741 tacaggaaaa tatacatcgc agggggttga cttttaccat ttcaccgcaa tggaatcaaa

4801 cttgttgaag agaatgttca caggcgcata cgctacaatg acccgattct tgctagcctt

4861 ttctcggtct tgcaaacaac cgccggcagc ttagtatata aatacacatg tacatacctc

4921 tctccgtatc ctcgtaatca ttttcttgta tttatcgtct tttcgctgta aaaactttat

4981 cacacttatc tcaaatacac ttattaaccg cttttactat tatcttctac gctgacagta

5041 atatcaaaca gtgacacata ttaaacacag tggtttcttt gcataaacac catcagcctc

5101 aagtcgtcaa gtaaagattt cgtgttcatg cagatagata acaatctata tgttgataat

5161 tagcgttgcc tcatcaatgc gagatccgtt taaccggacc ctagtgcact taccccacgt

5221 tcggtccact gtgtgccgaa catgctcctt cactatttta acatgtggaa ttaattctca

5281 tgtttgacag cttatcatcg aactctaaga ggtgatactt atttactgta aaactgtgac

5341 gataaaaccg gaaggaagaa taagaaaact cgaactgatc tataatgcct attttctgta

5401 aagagtttaa gctatgaaag cctcggcatt ttggccgctc ctaggtagtg ctttttttcc

5461 aaggacaaaa cagtttcttt ttcttgagca ggttttatgt ttcggtaatc ataaacaata

5521 aataaattat ttcatttatg tttaaaaata aaaaataaaa aagtatttta aatttttaaa

5581 aaagttgatt ataagcatgt gaccttttgc aagcaattaa attttgcaat ttgtgatttt

5641 aggcaaaagt tacaatttct ggctcgtgta atatatgtat gctaaagtga acttttacaa

5701 agtcgatatg gacttagtca aaagaaattt tcttaaaaat atatagcact agccaattta

5761 gcacttcttt atgagatata ttatagactt tattaagcca gatttgtgta ttatatgtat

5821 ttacccggcg aatcatggac atacattctg aaataggtaa tattctctat ggtgagacag

5881 catagataac ctaggataca agttaaaagc tagtactgtt ttgcagtaat ttttttcttt

5941 tttataagaa tgttaccacc taaataagtt ataaagtcaa tagttaagtt tgatatttga

6001 ttgtaaaata ccgtaatata tttgcatgat caaaaggctc aatgttgact agccagcatg

6061 tcaaccacta tattgatcac cgatatatgg acttccacac caactagtaa tatgacaata

6121 aattcaagat attcttcatg agaatggccc agcgatatat gcggtgtgaa ataccgcaca

6181 gatgcgtaag gagaaaatac cgcatcaggc gccattcgcc attcaggctg cgcaactgtt

6241 gggaagggcg atcggtgcgg gcctcttcgc tattacgcca gctggcgaaa gggggatgtg

6301 ctgcaaggcg attaagttgg gtaacgccag ggttttccca gtcacgacgt tgtaaaacga

6361 cggccagt

//

***Sequence PSMv4 Genbank format***

LOCUS Exported 6815 bp DNA circular SYN 27-AUG-2021

DEFINITION synthetic circular DNA.

ACCESSION .

VERSION .

KEYWORDS .

SOURCE synthetic DNA construct

ORGANISM synthetic DNA construct

REFERENCE 1 (bases 1 to 6815)

AUTHORS Hanna Alalam

TITLE Direct Submission

JOURNAL Exported Friday, Aug 27, 2021 from SnapGene Viewer 5.3.0

https://www.snapgene.com

FEATURES Location/Qualifiers

source 1..6815

/organism="recombinant plasmid"

/mol_type="other DNA"

promoter 7..500

/label=Met3p

CDS 507..995

/codon_start=1

/label=MazEF fusion-Mpro

/translation="MLIIEPVRKEPVFTLAELVNDITPENLHENIDWGEPKDKEVWGGV

KLQSGSMVSRYVPDMGDLIWVDFDPTKGSEQAGHRPAVVLSPFMYNNKTGMCLCVPCTT

QSKGYPFEVVLSGQERDGVALADQVKSIAWRARGATKKGTVAPEELQLIKAKINVLIG"

CDS 507..632

/codon_start=1

/label=mazE C terminal

/translation="MLIIEPVRKEPVFTLAELVNDITPENLHENIDWGEPKDKEVW"

misc_difference 633..638

/label=Flexibility linker

misc_feature 639..653

/label=SARS-CoV-2 Mpro cleavage site

misc_feature 654..659

/label=Flexibility linker

CDS 660..995

/codon_start=1

/label=mazF

/translation="MVSRYVPDMGDLIWVDFDPTKGSEQAGHRPAVVLSPFMYNNKTGM

CLCVPCTTQSKGYPFEVVLSGQERDGVALADQVKSIAWRARGATKKGTVAPEELQLIKA

KINVLIG"

terminator 1002..1263

/label=CYC1 terminator

/label=CYC1\terminator

rep_origin 1679..2272

/label=pMB1 ori

/label=pMB1\ori

CDS complement(2443..3303)

/codon_start=1

/label=ampR

/translation="MSIQHFRVALIPFFAAFCLPVFAHPETLVKVKDAEDQLGARVGYI

ELDLNSGKILESFRPEERFPMMSTFKVLLCGAVLSRIDAGQEQLGRRIHYSQNDLVEYS

PVTEKHLTDGMTVRELCSAAITMSDNTAANLLLTTIGGPKELTAFLHNMGDHVTRLDRW

EPELNEAIPNDERDTTMPVAMATTLRKLLTGELLTLASRQQLIDWMEADKVAGPLLRSA

LPAGWFIADKSGAGERGSRGIIAALGPDGKPSRIVVIYTTGSQATMDERNRQIAEIGAS

LIKHW"

CDS 3740..4542

/codon_start=1

/label=URA3

/translation="MSKATYKERAATHPSPVAAKLFNIMHEKQTNLCASLDVRTTKELL

ELVEALGPKICLLKTHVDILTDFSMEGTVKPLKALSAKYNFLLFEDRKFADIGNTVKLQ

YSAGVYRIAEWADITNAHGVVGPGIVSGLKQAAEEVTKEPRGLLMLAELSCKGSLSTGE

YTKGTVDIAKSDKDFVIGFIAQRDMGGRDEGYDWLIMTPGVGLDDKGDALGQQYRTVDD

VVSTGSDIIIVGRGLFAKGRDAKVEGERYRKAGWEAYLRRCGQQN"

rep_origin 4880..5624

/label=ARS1

rep_origin 5625..6601

/label=CEN 4

/label=CEN\4

ORIGIN

1 gaattcttta gtactaacag agacttttgt cacaactaca tataagtgta caaatatagt

61 acagatatga cacacttgta gcgccaacgc gcatcctacg gattgctgac agaaaaaaag

121 gtcacgtgac cagaaaagtc acgtgtaatt ttgtaactca ccgcattcta gcggtccctg

181 tcgtgcacac tgcactcaac accataaacc ttagcaacct ccaaaggaaa tcaccgtata

241 acaaagccac agttttacaa cttagtctct tatgaagtta cttaccaatg agaaatagag

301 gctctttctc gagaaatatg aatatggata tatatatata tatatatata tatatatata

361 tatatgtaaa cttggttctt ttttagcttg tgatctctag cttgggtctc tctctgtcgt

421 aacagttgtg atatcgtttc ttaacaattg aaaaggaact aagaaagtat aataataaca

481 agaataaagt ataattaaca ggatccatgt tgattattga accagttaga aaagaaccag

541 tttttacttt ggctgaattg gttaatgata ttactccaga aaatttgcat gaaaatattg

601 attggggtga accaaaagat aaagaagttt ggggtggtgt taaattgcaa tctggttcta

661 tggtttctag atatgttcca gatatgggtg atttgatttg ggttgatttt gatccaacta

721 aaggttctga gcaagctggt catagaccag ctgttgtttt gtctccattt atgtataata

781 ataaaactgg tatgtgtttg tgtgttccat gtactactca atctaaaggt tatccatttg

841 aagttgtttt gtctggtcaa gaaagagatg gtgttgcttt ggctgatcaa gttaaatcta

901 ttgcttggag agctagaggt gctactaaaa aaggtactgt tgctccagaa gaattgcaat

961 tgattaaagc taaaattaat gttttgattg gttaactgca ggagggccgc atcatgtaat

1021 tagttatgtc acgcttacat tcacgccctc cccccacatc cgctctaacc gaaaaggaag

1081 gagttagaca acctgaagtc taggtcccta tttatttttt tatagttatg ttagtattaa

1141 gaacgttatt tatatttcaa atttttcttt tttttctgta cagacgcgtg tacgcatgta

1201 acattatact gaaaaccttg cttgagaagg ttttgggacg ctcgaaggct ttaatttgcg

1261 gccaagcttg gcgtaatcat ggtcatagct gtttcctgtg tgaaattgtt atccgctcac

1321 aattccacac aacatacgag ccggaagcat aaagtgtaaa gcctggggtg cctaatgagt

1381 gagctaactc acattaattg cgttgcgctc actgcccgct ttccagtcgg gaaacctgtc

1441 gtgccagctg cattaatgaa tcggccaacg cgcggggaga ggcggtttgc gtattgggcg

1501 ctcttccgct tcctcgctca ctgactcgct gcgctcggtc gttcggctgc ggcgagcggt

1561 atcagctcac tcaaaggcgg taatacggtt atccacagaa tcaggggata acgcaggaaa

1621 gaacatgtga gcaaaaggcc agcaaaaggc caggaaccgt aaaaaggccg cgttgctggc

1681 gtttttccat aggctccgcc cccctgacga gcatcacaaa aatcgacgct caagtcagag

1741 gtggcgaaac ccgacaggac tataaagata ccaggcgttt ccccctggaa gctccctcgt

1801 gcgctctcct gttccgaccc tgccgcttac cggatacctg tccgcctttc tcccttcggg

1861 aagcgtggcg ctttctcata gctcacgctg taggtatctc agttcggtgt aggtcgttcg

1921 ctccaagctg ggctgtgtgc acgaaccccc cgttcagccc gaccgctgcg ccttatccgg

1981 taactatcgt cttgagtcca acccggtaag acacgactta tcgccactgg cagcagccac

2041 tggtaacagg attagcagag cgaggtatgt aggcggtgct acagagttct tgaagtggtg

2101 gcctaactac ggctacacta gaaggacagt atttggtatc tgcgctctgc tgaagccagt

2161 taccttcgga aaaagagttg gtagctcttg atccggcaaa caaaccaccg ctggtagcgg

2221 tggttttttt gtttgcaagc agcagattac gcgcagaaaa aaaggatctc aagaagatcc

2281 tttgatcttt tctacggggt ctgacgctca gtggaacgaa aactcacgtt aagggatttt

2341 ggtcatgaga ttatcaaaaa ggatcttcac ctagatcctt ttaaattaaa aatgaagttt

2401 taaatcaatc taaagtatat atgagtaaac ttggtctgac agttaccaat gcttaatcag

2461 tgaggcacct atctcagcga tctgtctatt tcgttcatcc atagttgcct gactccccgt

2521 cgtgtagata actacgatac gggagggctt accatctggc cccagtgctg caatgatacc

2581 gcgagaccca cgctcaccgg ctccagattt atcagcaata aaccagccag ccggaagggc

2641 cgagcgcaga agtggtcctg caactttatc cgcctccatc cagtctatta attgttgccg

2701 ggaagctaga gtaagtagtt cgccagttaa tagtttgcgc aacgttgttg ccattgctac

2761 aggcatcgtg gtgtcacgct cgtcgtttgg tatggcttca ttcagctccg gttcccaacg

2821 atcaaggcga gttacatgat cccccatgtt gtgcaaaaaa gcggttagct ccttcggtcc

2881 tccgatcgtt gtcagaagta agttggccgc agtgttatca ctcatggtta tggcagcact

2941 gcataattct cttactgtca tgccatccgt aagatgcttt tctgtgactg gtgagtactc

3001 aaccaagtca ttctgagaat agtgtatgcg gcgaccgagt tgctcttgcc cggcgtcaat

3061 acgggataat accgcgccac atagcagaac tttaaaagtg ctcatcattg gaaaacgttc

3121 ttcggggcga aaactctcaa ggatcttacc gctgttgaga tccagttcga tgtaacccac

3181 tcgtgcaccc aactgatctt cagcatcttt tactttcacc agcgtttctg ggtgagcaaa

3241 aacaggaagg caaaatgccg caaaaaaggg aataagggcg acacggaaat gttgaatact

3301 catactcttc ctttttcaat attattgaag catttatcag ggttattgtc tcatgagcgg

3361 atacatattt gaatgtattt agaaaaataa acaaataggg gttccgcgca catttccccg

3421 aaaagtgcca cctgacgtct aagaaaccat tattatcatg acattaacct ataaaaatag

3481 gcgtatcacg aggccctttc gtcttcaaga attagctttt caattcaatt catcattttt

3541 tttttattct tttttttgat ttcggtttct ttgaaatttt tttgattcgg taatctccga

3601 acagaaggaa gaacgaagga aggagcacag acttagattg gtatatatac gcatatgtag

3661 tgttgaagaa acatgaaatt gcccagtatt cttaacccaa ctgcacagaa caaaaacatg

3721 caggaaacga agataaatca tgtcgaaagc tacatataag gaacgtgctg ctactcatcc

3781 tagtcctgtt gctgccaagc tatttaatat catgcacgaa aagcaaacaa acttgtgtgc

3841 ttcattggat gttcgtacca ccaaggaatt actggagtta gttgaagcat taggtcccaa

3901 aatttgttta ctaaaaacac atgtggatat cttgactgat ttttccatgg agggcacagt

3961 taagccgcta aaggcattat ccgccaagta caatttttta ctcttcgaag acagaaaatt

4021 tgctgacatt ggtaatacag tcaaattgca gtactctgcg ggtgtataca gaatagcaga

4081 atgggcagac attacgaatg cacacggtgt ggtgggccca ggtattgtta gcggtttgaa

4141 gcaggcggca gaagaagtaa caaaggaacc tagaggcctt ttgatgttag cagaattgtc

4201 atgcaagggc tccctatcta ctggagaata tactaagggt actgttgaca ttgcgaagag

4261 cgacaaagat tttgttatcg gctttattgc tcaaagagac atgggtggaa gagatgaagg

4321 ttacgattgg ttgattatga cacccggtgt gggtttagat gacaagggag acgcattggg

4381 tcaacagtat agaaccgtgg atgatgtggt ctctacagga tctgacatta ttattgttgg

4441 aagaggacta tttgcaaagg gaagggatgc taaggtagag ggtgaacgtt acagaaaagc

4501 aggctgggaa gcatatttga gaagatgcgg ccagcaaaac taaaaaactg tattataagt

4561 aaatgcatgt atactaaact cacaaattag agcttcaatt taattatatc agttattacc

4621 caattctcat gtttgacagc ttatcatcga tcgtccaact gcatggagat gagtcgtggc

4681 aagaatacca agagttcctc ggtttgccag ttattaaaag actcgtattt ccaaaagact

4741 gcaacatact actcagtgca gcttcacaga aacctcattc gtttattccc ttgtttgatt

4801 cagaagcagg tgggacaggt gaacttttgg attggaactc gatttctgac tgggttggaa

4861 ggcaagagag ccccgagagc ttacatttta tgttagctgg tggactgacg ccagaaaatg

4921 ttggtgatgc gcttagatta aatggcgtta ttggtgttga tgtaagcgga ggtgtggaga

4981 caaatggtgt aaaagactct aacaaaatag caaatttcgt caaaaatgct aagaaatagg

5041 ttattactga gtagtattta tttaagtatt gtttgtgcac ttgcctgcaa gccttttgaa

5101 aagcaagcat aaaagatcta aacataaaat ctgtaaaata acaagatgta aagataatgc

5161 taaatcattt ggctttttga ttgattgtac aggaaaatat acatcgcagg gggttgactt

5221 ttaccatttc accgcaatgg aatcaaactt gttgaagaga atgttcacag gcgcatacgc

5281 tacaatgacc cgattcttgc tagccttttc tcggtcttgc aaacaaccgc cggcagctta

5341 gtatataaat acacatgtac atacctctct ccgtatcctc gtaatcattt tcttgtattt

5401 atcgtctttt cgctgtaaaa actttatcac acttatctca aatacactta ttaaccgctt

5461 ttactattat cttctacgct gacagtaata tcaaacagtg acacatatta aacacagtgg

5521 tttctttgca taaacaccat cagcctcaag tcgtcaagta aagatttcgt gttcatgcag

5581 atagataaca atctatatgt tgataattag cgttgcctca tcaatgcgag atccgtttaa

5641 ccggacccta gtgcacttac cccacgttcg gtccactgtg tgccgaacat gctccttcac

5701 tattttaaca tgtggaatta attctcatgt ttgacagctt atcatcgaac tctaagaggt

5761 gatacttatt tactgtaaaa ctgtgacgat aaaaccggaa ggaagaataa gaaaactcga

5821 actgatctat aatgcctatt ttctgtaaag agtttaagct atgaaagcct cggcattttg

5881 gccgctccta ggtagtgctt tttttccaag gacaaaacag tttctttttc ttgagcaggt

5941 tttatgtttc ggtaatcata aacaataaat aaattatttc atttatgttt aaaaataaaa

6001 aataaaaaag tattttaaat ttttaaaaaa gttgattata agcatgtgac cttttgcaag

6061 caattaaatt ttgcaatttg tgattttagg caaaagttac aatttctggc tcgtgtaata

6121 tatgtatgct aaagtgaact tttacaaagt cgatatggac ttagtcaaaa gaaattttct

6181 taaaaatata tagcactagc caatttagca cttctttatg agatatatta tagactttat

6241 taagccagat ttgtgtatta tatgtattta cccggcgaat catggacata cattctgaaa

6301 taggtaatat tctctatggt gagacagcat agataaccta ggatacaagt taaaagctag

6361 tactgttttg cagtaatttt tttctttttt ataagaatgt taccacctaa ataagttata

6421 aagtcaatag ttaagtttga tatttgattg taaaataccg taatatattt gcatgatcaa

6481 aaggctcaat gttgactagc cagcatgtca accactatat tgatcaccga tatatggact

6541 tccacaccaa ctagtaatat gacaataaat tcaagatatt cttcatgaga atggcccagc

6601 gatatatgcg gtgtgaaata ccgcacagat gcgtaaggag aaaataccgc atcaggcgcc

6661 attcgccatt caggctgcgc aactgttggg aagggcgatc ggtgcgggcc tcttcgctat

6721 tacgccagct ggcgaaaggg ggatgtgctg caaggcgatt aagttgggta acgccagggt

6781 tttcccagtc acgacgttgt aaaacgacgg ccagt

//
