# Supplementary figures and images for: A Genetic Trap in Yeast for Inhibitors of SARS-CoV-2 Main Protease
Source: mSystems. 2021 Nov 23;6(6):e01087-21. doi: 10.1128/mSystems.01087-21 (PMC8609969; doi:10.1128/mSystems.01087-21)

**Mpro + MazEF-GAL**

**Fig. S1**

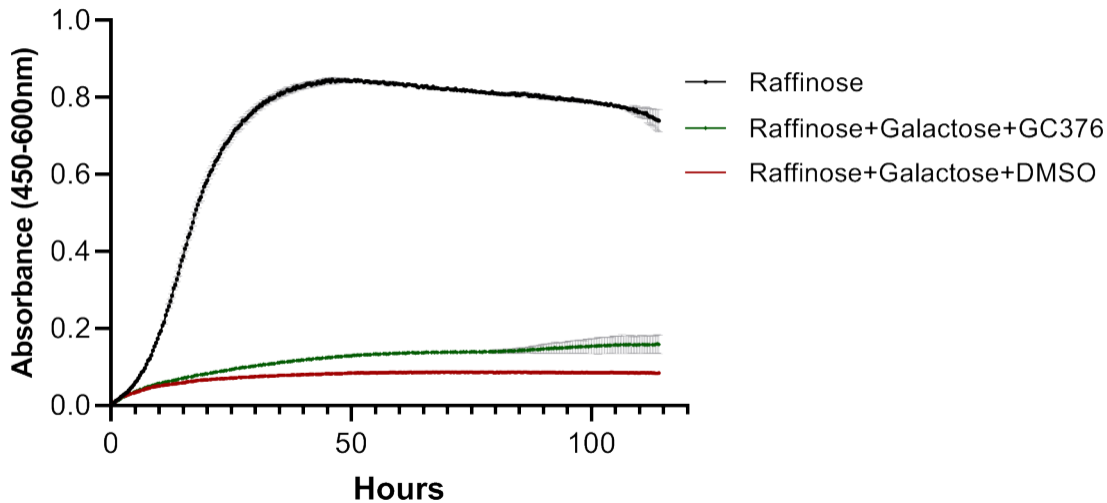

Supplement: FIG S1 [file msystems.01087-21-sf001.pdf]

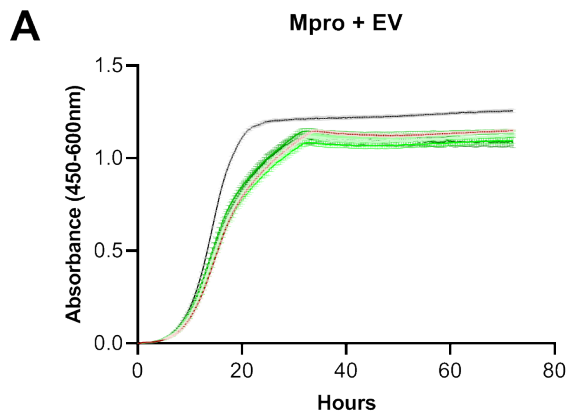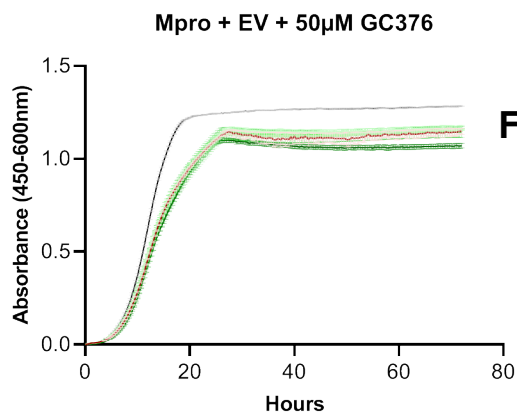

**Fig. S2**

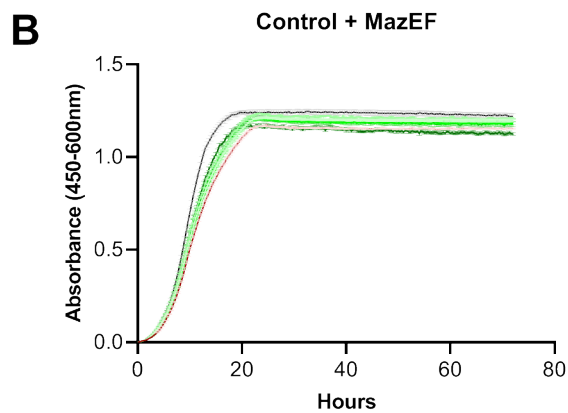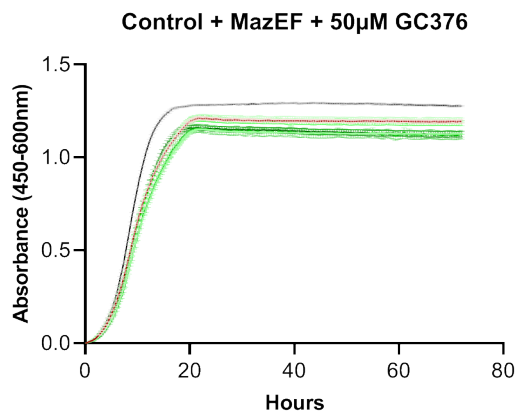

Met ( $\mu$ M)

— 350

— 120

— 30

— 15

— 7.5

— 3.75

— 0

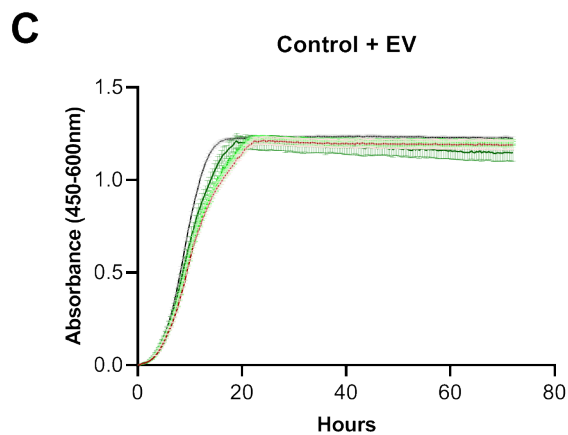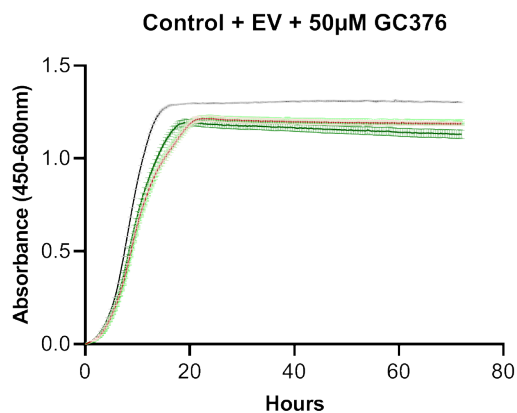

Supplement: FIG S2 [file msystems.01087-21-sf002.pdf]

**Fig. S3**

**A**

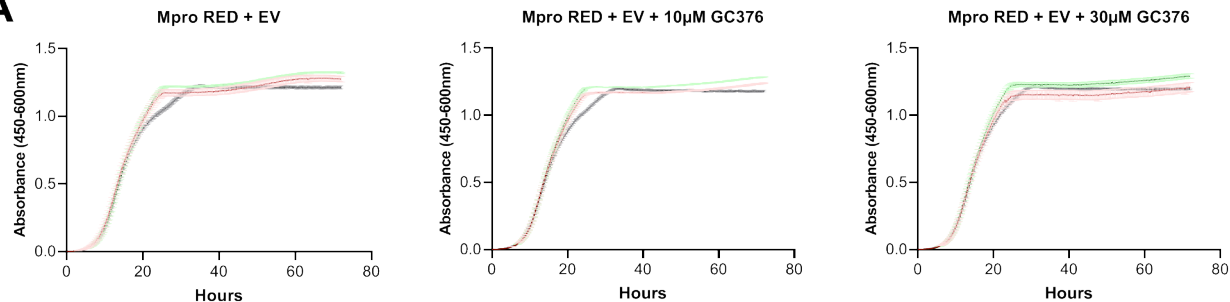

**B**

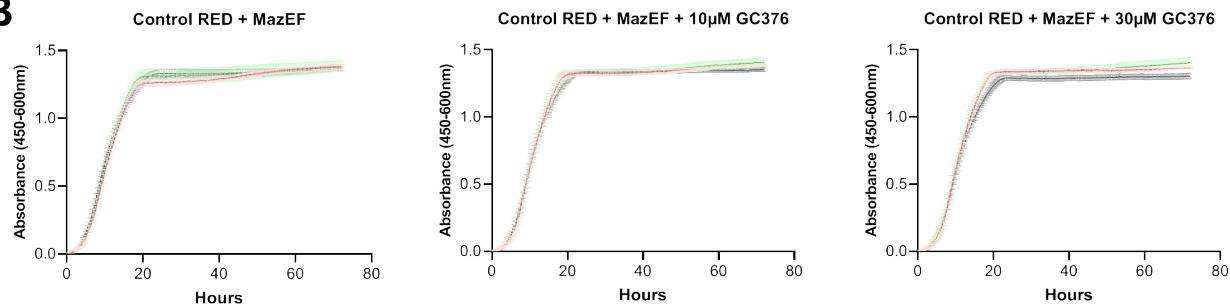

**Met ( $\mu$ M)**

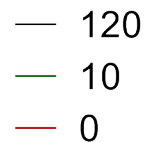

**C**

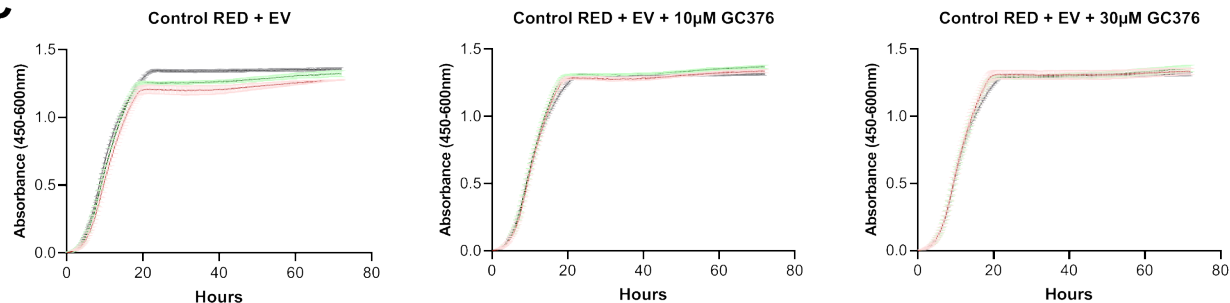

Supplement: FIG S3 [file msystems.01087-21-sf003.pdf]

**Fig. S4**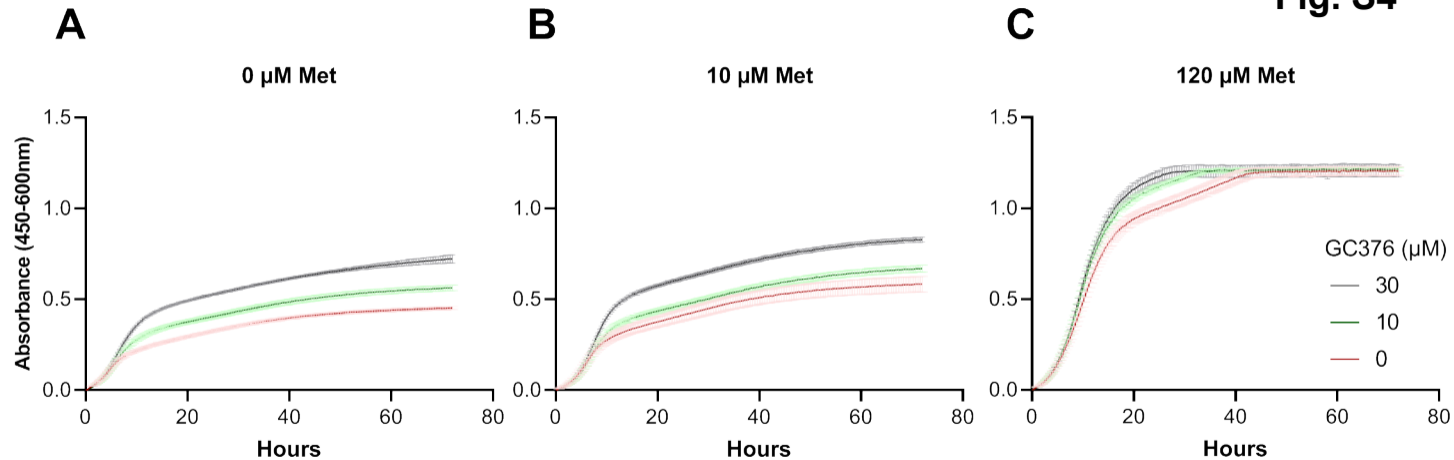

Supplement: FIG S4 [file msystems.01087-21-sf004.pdf]

# Mpro vs Control

Fig. S5

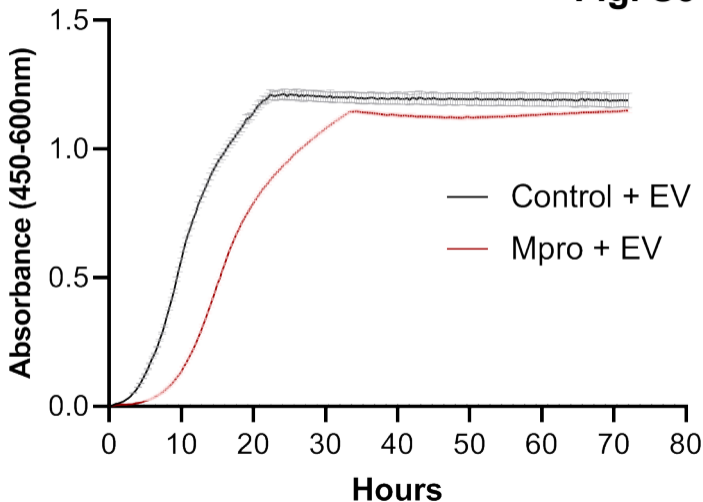

Supplement: FIG S5 [file msystems.01087-21-sf005.pdf]
