# Supplementary material for: A Genetic Trap in Yeast for Inhibitors of SARS-CoV-2 Main Protease
Source: mSystems. 2021 Nov 23;6(6):e01087-21. doi: 10.1128/mSystems.01087-21 (PMC8609969; doi:10.1128/mSystems.01087-21)
Supplement: TABLE S1 [file msystems.01087-21-st001.docx]

**Table S1** Primers used for strain or plasmid construction

For strain construction

| Primer | Sequence 5’ to 3’ | Comment |
| --- | --- | --- |
| PDR1A | ACCCTAAATGGAGTTTCTTTTCTTG | Deletion check *PDR1* |
| PDR1D | CCCATAAGAAATACACCTCATGGTA | Deletion check *PDR1* |
| PDR3A | TTCACTTTTCATTTTCTTCCAGAAC | Deletion check *PDR3* |
| PDR3D | AATATCTACTGAACAGCTGCATTCC | Deletion check *PDR3* |
| SNQ2A | CACCACTTTTTATGCTTGTATATGCT | Deletion check *SNQ2* |
| SNQ2D | GTTTAGCTTGTACTTGCAACACCTT | Deletion check *SNQ2* |
| Met17_pdr3_int_F | **CCATTATTTCCACGACAACTGCATTTCACTTTTCATTTTCTTCCAGAAC***GGATCC*GAAACCTCCATCATCCTCTT | Integration of *MET17* into *PDR3* locus/ligation of *MET17* to Mpro  (*Bam* HI: *Italics*, recombination site: **Bold**) |
| Met17_pdr3_int_R | **AATTGTTGAGGCTATAATTACATATAATATCTACTGAACAGCTGCATT**CC*GGATCC*TTATTTTTTGCTTTTTCTCTTGAGG | Integration of *MET17* into PDR3 locus/ligation of *MET17* to Mpro/ Integration of *MET17*-Mpro into *PDR3*  (*Bam* HI: *Italics*, recombination site: **Bold**) |
| Flurotag_marker_F | ATGACAGAGCAGAAAGCC | mCherry tag integration into *HIS3* |
| Flurotag_marker_R | TATACACATGTATATATATCGTATGCTGCAGCTTTAAATAATCGGTGTCAGGATCCGTTAGAATCATTTTGAAT | mCherry tag integration into *HIS3* |
| Mpro_met17_pdr3_int_F | **CCATTATTTCCACGACAACTGCATTTCACTTTTCATTTTCTTCCAGAAC**GGACCATTGCTGAATCACAAA | Integration of *MET17*-Mpro into *PDR3* locus  (Recombination site: **Bold**) |

For plasmid construction

| Primer | Sequence 5’ to 3’ | Comment |
| --- | --- | --- |
| EcoRI_GAL1p_F | GGTAC*GAATTC*AGTACGGATTAGAAGCCGC | Amplification of *GAL1* promoter. Restriction sites are *italicized*. |
| BglII_GAL1p_R | CTCCG*AGATCT*GTTTTTTCTCCTTGACGTTAAAGTAT | Amplification of *GAL1* promoter. Restriction sites are *italicized*. |
| EcoRI_ MET3p _F | GGTAC*GAATTC*TTTAGTACTAACAGAGACTTTTGTCACA | Amplification of *MET3* promoter. Restriction sites are *italicized*. |
| BamHI_ MET3p _R | CTCCG*GGATCC*TGTTAATTATACTTTATTCTTGTTATTATTATACTTTCTTAGT | Amplification of *MET3* promoter. Restriction sites are *italicized*. |
